# Assessment of Commercially Available Immunoassays to Measure Glucocorticoid Metabolites in African Grey Parrot (*Psittacus Erithacus*) Droppings: A Ready Tool for Non-Invasive Monitoring of Stress

**DOI:** 10.3390/ani8070105

**Published:** 2018-06-28

**Authors:** Cécile Bienboire-Frosini, Muriel Alnot-Perronin, Camille Chabaud, Pietro Asproni, Céline Lafont-Lecuelle, Alessandro Cozzi, Patrick Pageat

**Affiliations:** 1 Department of Physiological and Behavioral Mechanisms of Adaptation (DMPCA), IRSEA (Research Institute in Semiochemistry and Applied Ethology), 84400 Apt, France; c.chabaud@group-irsea.com; 2 Clinical Ethology and Animal Welfare Centre (CECBA), IRSEA (Research Institute in Semiochemistry and Applied Ethology), 84400 Apt, France; malnot@orange.fr; 3 Department of Cellular Processes and Sustainable Interactions (DPCID), IRSEA (Research Institute in Semiochemistry and Applied Ethology), 84400 Apt, France; p.asproni@group-irsea.com; 4 Statistical Analyses Department, IRSEA (Research Institute in Semiochemistry and Applied Ethology), 84400 Apt, France; c.lecuelle@group-irsea.com; 5 Research and Education Board, IRSEA (Research Institute in Semiochemistry and Applied Ethology), 84400 Apt, France; a.cozzi@group-irsea.com (A.C.); p.pageat@group-irsea.com (P.P.)

**Keywords:** analytical validation, captivity, enzyme immunoassay, Fecal Glucorticoid Metabolites, Heterophil:Lymphocyte Ratio, non-invasive stress measurement, parrot, welfare monitoring

## Abstract

**Simple Summary:**

The African Grey Parrot is a very popular bird commonly found in zoological collections. However, captivity can prevent it from meeting its natural needs and so become an ongoing stressor, leading sometimes to clinical and/or behavioral disorders. Non-invasive forms of stress assessment are of definite interest for monitoring welfare in captive bird populations. One notable stress outcome is the excretion of glucocorticoid metabolites (from the stress hormone corticosterone) in droppings. The aim of this study was to carefully assess methods of glucocorticoid metabolites extraction and measurement in droppings from African Grey Parrots. Several extraction and enzyme immunoassays procedures were tested, based on the evaluation of analytical quality parameters and biological relevance. The best procedure was found to be a combination of a 60% methanol extraction with the use of a commercial corticosterone enzyme immunoassay. To determine whether this method was suitable for assessing different stress levels, a significant correlation with another reliable stress marker in birds, the Heterophil: Lymphocyte Ratio, was evidenced. This method can thus be used to evaluate stress in African Gray Parrots in a non-invasive way and help to monitor their welfare in zoo populations for instance.

**Abstract:**

Despite being undomesticated, African Grey Parrots (*Psittacus erithacus*) are commonly found in captivity, in zoos or as pets. Captivity can be an ongoing stressor. Non-invasive glucocorticoid metabolites (GCM) measurements from bird droppings are of interest for assessing stress but require careful evaluation in each newly studied species. This study describes the assessment of such methods for *Psittacus erithacus* to provide tools for evaluating stress and monitoring welfare. We evaluated 12 method combinations of GCM extraction and enzyme immunoassay (EIA) from a pool of African Grey Parrot droppings, through the validation of several analytical parameters. Then, Heterophil: Lymphocyte Ratios (HLR), another reliable stress marker, were determined and correlated to individual dropping GCM concentrations for 29 birds to determine whether the method is biologically relevant. We found that the best procedure to measure GCM in African Grey Parrot droppings is a combination of 60% methanol extraction measured using a Corticosterone EIA kit (Cayman Chemical Company) from fresh or dry droppings. The establishment of a significant correlation (Pearson coefficient correlation = 0.48; *p* = 0.0082) between HLR and GCM in the studied population confirmed the method biological relevance. This method can thus be applied to assess stress in *Psittacus erithacus* and support welfare monitoring in zoo populations.

## 1. Introduction

The African Grey Parrot (*Psittacus erithacus*) belongs to the order Psittaciformes, which includes very different species (~356) from different biotopes with very specific needs [1]. It has recently been up-listed to endangered on the International Union for Conservation of Nature’s Red List of Threatened Species [2]. The major threat to the African Grey Parrot species arises from its status as a very popular avian pet [3], prized for its longevity, intelligence, and unparalleled ability to imitate human speech and other sounds [4,5]. However, they are not truly domesticated animals, because most are directly removed from the wild, due to the remaining illegal trade, or bred for only one or two generations [6,7]. Parrot breeding for the pet trade can also be problematic because it is predominated by hand-reared birds that may exhibit several behavioral abnormalities, notably due to parental separation [8,9,10]. African Grey Parrots are also increasingly common in zoological collections [11], but their intelligent and social nature and sensitivity combined with the difficulty of meeting their natural needs in captivity make them particularly vulnerable to stress [7,12,13]. Overall, captivity may be an ongoing stressor which can threaten the welfare of these birds [6,7,13,14].

Indeed, chronic stress can increase vulnerability to disease and predisposition to injury as well as being the root of various abnormal behaviors [13,15]. In *Psittacus erithacus*, self-destructive behaviors, stereotypy, eating disorders, attachment disorders, phobias, aggression, and inappropriate vocalizations are some of the most common symptoms observed and can be related to stress or poor welfare [3,13,16]. For instance, the feather damaging behavior is a very common issue related to self-destructive behaviors and can lead in some cases to serious medical problems [17,18]. These behavioral disorders are frequently assessed based on clinical experience [19], but the diagnosis is often challenging because many signs and symptoms are scarce and nonspecific [20]. Despite several investigations, the etiology of such abnormal behaviors is complex, probably multifactorial, and requires further investigation [3,18]. Although zoos have conservation goals, this does not necessarily mean welfare is always optimum and there can still be captivity-related stress, evidenced by abnormal behaviors seen in zoo-housed birds [9,12]. Being able to monitor stress levels in captive animals could facilitate diagnosis and shed light on the underlying causes of such abnormal behaviors. Furthermore, having reliable monitoring tools is of the utmost importance for the birds’ welfare in order to prevent behavioral disorders before clinical symptoms appear. Hence, stress monitoring of African Grey Parrots in zoos or ornithological parks is crucial to assessing their welfare and improving their management and husbandry in the long term [12,21].

In birds, stressful situations lead to the activation of the Hypothalamo-Pituitary-Axis (HPA) and to the release of cortisol and corticosterone into the blood by the adrenal gland. Of the two hormones, corticosterone is predominant in birds and is thereafter metabolized and excreted in droppings [22]. The non-invasive measurement of glucocorticoid metabolites (GCM) from excreta (urine, feces, droppings) is now widely used to assess stress/welfare issues in various species, including zoo animals [23,24]. This method is particularly interesting in wild or captive endangered species since non-invasive samples can often be obtained without disturbing, and thus further stressing, the animal through manipulation [25,26,27]. GCM provides a reliable measure of the animal’s “stress status” since levels of circulating hormone are integrated over a longer period, thus capable of reflecting chronic stress. Conversely, an isolated blood sample may not reflect long-term stress hormone levels due to the pulsatile secretion pattern of glucocorticoids in the blood [26]. In addition, blood sampling can be problematic if the collection procedure itself induces a stress response. Moreover, a correlation between plasma glucocorticoid level and its fecal metabolites has been demonstrated in several animals [28,29]. Corticosterone assays can also be conducted on bird feathers as another non-invasive, long-term measurement technique [30], but corticosterone concentration in feathers was found to be rarely correlated with plasma corticosterone and may not even be correlated with behaviors and/or treatments related to putative stress states [31,32,33]). Indeed, Bortolotti [34] highlighted the flaws and difficulties of interpreting of feather corticosterone concentration, in addition to the ethical considerations if the feathers are plucked [35]. 

Regarding the biological effect of the stress response, it is also important to consider not only the magnitude of the peripheric glucocorticoid release but also its duration, i.e., the integrated response. In excreta samples, hormone levels are integrated over a certain time period and thus represent cumulative secretion. In the case of glucocorticoid hormone levels, these measures can therefore reflect the integrated response to stress and provide a more accurate assessment of long-term glucocorticoid levels and long-term stressors [22]. For all the reasons stated above, GCM measurements in droppings are of utmost interest in evaluating stress/welfare in *Psittacus erithacus*. But because clear differences regarding the metabolism and excretion of GCM exist between species and sexes, a careful evaluation of the method used to assay GCM in feces or droppings is required for each species and sex investigated, as highlighted by some authors [24,26,36,37]. Some authors [38] have already described a method to assess GCM in *Psittacus erithacus* droppings in association with a specific behavioral disorder (feather damaging behavior), but without a clear link to a stress-related condition. Here, our approach was to provide a detailed analytical assessment of a GCM measurement method from readily available immunoassays and clearly correlate with a stressful state in African Grey Parrots as a part of a general welfare evaluation independent of a specific clinical/behavioral disorder.

We first assessed several protocols of dropping handling in different combinations of GCM extraction and immunoassay methods. After the selection of the best global procedure, we finalized the analytical validation to fulfil usual performance criteria. We completed the method assessment by showing its biological relevancy thanks to the establishment of correlations between GCM levels in droppings and a reliable stress biomarker, the Heterophil: Lymphocyte Ratio (HLR) [39,40], within the studied population to demonstrate the relevance of this method in assessing stress levels in *Psittacus erithacus*. The method described could for instance then be used to monitor welfare in zoo-housed African Grey Parrots. 

## 2. Materials and Methods

### 2.1. Birds

The study population comprised 33 African Grey Parrots (*Psittacus erithacus*) (15 females; 18 males) with no visible sign of pre-existing pathology. The birds were located at a rescue center managed by a non-profit institution (Association de Sauvegarde et d’Accueil des Perroquets—ASAP, Raizeux, France). The ASAP center is approved by French authorities and welcomes parrots rescued from pound services or illegal traffic. Because ASAP parrots are all rescued and not born at the institution, the exact age of the parrots involved in this study cannot be known, but they all are sexually mature adults. All the parrots involved in this study had spent at least one year in the rescue center. Newly welcomed parrots were thus excluded as they may be still being adapting to the rescue center environment. The parrots were housed alone (*n* = 18) or in small groups (2–4 individuals, *n* = 15 in total) in small to large aviaries. They were fed once a day in the morning with seeds, fresh fruits, and vegetables and had access to tap water ad libitum. 

All procedures in this study were performed in accordance with French and European legislation and with the ethical standards of the IRSEA Ethics Committee C2EA125, which evaluated and approved them under the no.: CE_201501_01.

### 2.2. Sample Collection

In the first step of sampling, for the methods analytical assessment and selection, droppings from 18 African Grey Parrots of both sexes, housed alone, were collected once, directly from their regular cages. From the practical point of view, it was indeed simpler to collect droppings from cages housing only one parrot. In the second step of sampling, in order to investigate the biological relevancy of the method by correlating the GCM concentrations of individual droppings with HLR in a larger population, the parrots’ droppings were collected according to the following procedure, which combined social isolation, individual droppings collection, and blood sampling. For this droppings collection, 33 African Grey Parrots were individually caught by hand from their regular cages and placed for 30 min in the morning (9 a.m.–12 p.m.) in individual cages of 60 × 100 × 60 cm (height, width, and depth, respectively) located in a room outside of their living areas (thus preventing them from witnessing the procedures being carried out with other animals) where they could defecate on a clean aluminum foil lining the cage floor. The handling of each parrot was performed by the same two manipulators during the study. The isolation period of 30 min is described in the literature [41,42] as sufficient to observe defecation, although two birds did not produce droppings during the 30-min period: consequently, the total number of collected droppings is 31. The sample collection carried out in the second step was performed once per parrot. Because glucocorticoid release follows a circadian rhythm, we tested four parrots each day in order to maintain the 9 a.m.–12 p.m time window for sampling. All the aluminum foils containing the droppings were folded, identified and stored immediately at −20 °C to prevent sample degradation. Once the parrots had been taken out the cage, a blood smear was obtained with a drop of blood collected from the brachial vein from each of the 33 parrots. A hemostatic agent (Aluspray^®^; Vetoquinol, Lure, France) was applied to stop the bleeding. 

### 2.3. HLR Count

Blood smears were stained using a May-Grunwald-Giemsa commercial kit (RAL555, RAL Diagnostic, Martignac, France) according to manufacturer’s instructions and air-dried before microscopic observation. A total of 100 lymphocyte and heterophil cells were counted at ×250 magnification, and the HLR was calculated for all 33 individuals by dividing the number of heterophils by the number of lymphocytes [43].

### 2.4. Methods Assessment and Selection

Several combinations of methods to extract and assay GCM were evaluated: (i) two pre-extraction treatments of droppings (fresh vs. dry), (ii) two extraction buffers (60% ethanol vs. 60% methanol), and (iii) three commercially available enzyme immunoassay (EIA) kits initially designed for assaying corticosterone (from Enzo Life Sciences—ELS, Cayman Chemical Company—CCC, and ImmunoDiagnostic Systems—IDS). The recognition of GCM arises from inherent cross-reactions with the antibody; these cross-reactions should be as numerous as possible in order to recognize a maximum number of metabolites [44]. These three commercial kits were chosen for their cross-reactivities with known GCM in birds [45,46], as described by the providers (Table 1).

To select the best possible combination of procedures, several parameters were assessed in the assays: (i) the maximum binding (Bo) and the blank signal (Blk), reflecting the maximum quantification capability and the background absorbance caused by the kit’s reagents, respectively (both are key parameters of immunoassays, since defining their dynamic ranges of measurements); (ii) measures of a “spiked blank extract” in the EIA to assess the recovery of a known amount of corticosterone alone in the extraction buffer (standard corticosterone was used because of the impossibility of using “standard” GCM from parrot droppings as their chemical natures can be variable); (iii) measures of corticosterone recovery after spiking with a known amount of standard corticosterone the droppings before undergoing the global procedures of extraction and EIA (again, standard corticosterone was used by default to evaluate the extraction efficiency of this molecule solely, and the authors are well aware that the unmetabolized corticosterone recovery measured may not correspond with the actual recovery of GCM: this is why we refer to this measure as “virtual recovery”); (iv) measures of the relative accuracy of the global procedure by performing identical assessments using a known amount of droppings and twice this amount in order to test the linearity of GCM concentration and possibly the mass/volume effect; and (v) the linearity under dilution tested on two working dilutions of ½ and ¼ to confirm the absence of a matrix effect in actual dropping samples.

To compare these 12 combinations of methods, a sample was made by pooling 18 droppings gathered during the first step of the sample collection: the samples were quickly defrosted (5–10 min) to avoid any further bacterial degradation of the metabolites, then thoroughly homogenized, and undigested material was removed to ensure reproducibility. It was necessary to pool several droppings to carry out the analytical tests on one identical sample and to reach a mass of droppings sufficient enough to perform all the analytical tests carried out on the 12 combinations of methods. This pool was weighed and divided into three subsequent samples of defined weights: S1, S2 (whose mass is half that of S1), and S3 (whose mass is equal to S2 but spiked with 0.62 ng of exogenous corticosterone). Exogenous corticosterone was made by combining the corticosterone standards of the three kits. Each of the three samples underwent one method combination and was consequently divided into four parts of equal weight so that each was involved in one pre-extraction treatment and one extraction method (Table 2).

For the pre-extraction treatment, the samples could be used fresh immediately after defrosting or dried after an overnight incubation at 95 °C (complete dryness was verified by monitoring dry weights). 

GCM were then extracted by suspending the dropping samples in the extraction buffer (60% ethanol or 60% methanol) at a proportion of 1 mL/0.1 g of droppings (fresh or dry) followed by a thorough vortexing (30 min; room temperature). The suspensions were then centrifuged (5 min; 3000 g), and the supernatants were recovered and stored at −20 °C until the assay.

The 12 extracted samples were then analyzed with each of the three EIA kits (Enzo Life Sciences, Villeurbanne, France; Cayman Chemical Company, Ann Harbor, USA; ImmunoDiagnostic Systems, Pouilly-en-Aixois, France) at two working dilutions in kit assay buffers (1:2 and 1:4) following the manufacturer’s instructions. Optical density (OD) was measured at a wavelength of 405 nm (ELS EIA kit), 412 nm (CCC EIA kit), and 450 nm (IDS EIA kit) on an automatic plate reader (EPOCH Biotek Instruments, Colmar, France). 

Using the formula computation recommended in Palme et al. [47], GCM concentrations are reported in ng of GCM per g of droppings to facilitate the comparisons of % recovery between all the results, regardless of pre-extraction treatment (dry or fresh) and weights variation. 

### 2.5. Analytical Validation of the Selected Procedure

The analytical validation of the selected method was carried out using a series of experiments. Parallelism was studied between two-fold dilutions in the kit assay buffer (1:2 to 1:128) of three pooled extracts (treated in accordance with the previously selected procedure) and the standard curve to check the absence of interferences and the dose–response relationship. To monitor precision, low (~70% binding), medium (~50% binding), and high (~10% binding) quality control (QC) samples were run in five replicates after a 1:2 dilution and a global precision profile including all the samples from the entire study was established.

### 2.6. Biological Assessment of the Procedure and Statistical Procedures

To evaluate the biological relevance of the GCM assay in African Grey Parrot droppings, the relationship between individual dropping GCM concentration and HLR was investigated using the samples collected during the second step. To assess the robustness of the method, the impact of sex and dropping masses on GCM levels were also considered.

Data analysis was performed using SAS 9.4 software Copyright © 2002–2012 by SAS Institute Inc., Cary, NC, USA. The significance threshold was classically fixed at 5%. Data analysis was thus carried out using a Student t test with the help of proc *t* test procedure or Wilcoxon Two-Sample Test using npar1way procedure in SAS 9.4 software depending on normality and variances (homogeneity of variances will be verified using Fisher test using *t* test procedure also). The calculation of Pearson correlation coefficient (*r*) was performed using the proc corr procedure. According to Martin and Bateson [48], *r* < 0.2 is considered a slight, almost negligible correlation, *r* = 0.2–0.4 is considered a low correlation (small relationship), *r* = 0.4–0.7 is considered as a moderate correlation (substantial relationship), *r* = 0.7–0.9 is considered as a high correlation (marked relationship), and *r* = 0.9–1.0 is considered a very high correlation (very dependable relationship).

## 3. Results

### 3.1. Methods Assessment and Selection

#### 3.1.1. Extraction Buffers Influence on EIA Key Parameters

We aimed to assess the possible influence of the extraction buffers in two working dilutions on the dynamic range of each commercial assay. Table 3 shows Bo and Blk OD measured in two dilutions of 60% ethanol and 60% methanol in the tested EIA kits. Under normal conditions of use, the highest Bo signal was measured in the CCC EIA kit (OD = 1.391, producer, city, country). When measured in extraction buffers at two dilutions, the maximum OD value for Bo was obtained with the CCC EIA kit in 60% methanol diluted 1:4. Slightly lower values were found in 60% methanol diluted 1:2 with the CCC EIA kit and with the IDS kit, irrespective of the working dilutions. Of note, in every commercial kit, the highest Bo values were observed in the 60% methanol extraction buffer. The Blk OD values were similar for both extraction buffers and under normal conditions of use in every kit. 

#### 3.1.2. Corticosterone Recovery in Extraction Buffers

We also examined the effect of extraction buffers on the antibody-binding of the molecule of corticosterone alone by measuring a “spiked blank extract.” Recoveries were highly variable among the tested conditions, with a broad range of 11 to 258 % (Table 4). The best % recovery (101 %) was found when using 60% methanol diluted 1:2 with the CCC EIA kit. Acceptable % recoveries were also obtained using 60% methanol diluted 1:4 with the CCC EIA kit (88%) and using 60% ethanol diluted 1:4 with the IDS EIA kit (89%). Conversely, unsatisfactory results were obtained when using ELS EIA kit, irrespective of the extraction buffers and working dilution, and the CCC EIA kit in 60% ethanol, with % recoveries out of the acceptable range of 80–120% for immunoassays [49].

#### 3.1.3. Corticosterone “Virtual Recovery” in African Grey Parrots’ Droppings

The corticosterone “virtual recovery” in droppings was measured by comparing the GCM concentrations found in samples S2 and S3 under all tested conditions. As shown in Table 5, all but four of the % recoveries under the tested conditions fell within the acceptable range of 80–120%. Looking at the methods individually, (i) the pre-extraction treatments displayed similar mean % recoveries with 103.1% vs. 100.2% for dry vs. fresh feces, (ii) the extraction buffer caused slightly more differences in the mean % recovery (94.9% for 60% ethanol vs. 107.7% for 60% methanol), and (iii) the EIA kits presented comparable mean % recovery with less than 5% of bias (96.6%, 103.6%, and 104.8% for ELS, CCC, and IDS respectively). Of note, the raw GCM concentrations values in the same sample (S2 or S3) differed according to the commercial kit used, with approximately a 1.6-fold increase in measures from ELS and CCC and a 10-fold increase in measures from ELS and IDS. Particularly, for the non-spiked sample (S2), the baseline GCM concentrations showed a wide variation depending on the EIA used: 48.5 ± 17.6 ng/g, 81.4 ± 31.2 ng/g, and 499.6 ± 221.9 ng/g for ELS, CCC, and IDS, respectively. 

#### 3.1.4. Relative Accuracy of Each Methods Combination 

The relative accuracy was estimated by comparing the GCM concentrations found in samples S1 and S2, whose mass is half that of S1, under all tested conditions (Table 6). Satisfactory relative accuracies (% recovery within 80–120% so that the bias < 20%) were found under all tested conditions. Taking each individual method into account, the pre-extraction treatments led to nearly equal mean % recoveries (100.1% for dry vs. 99.8% for fresh feces); the extraction buffers resulted in 96.6% and 102.8% mean recoveries for 60% ethanol and 60% methanol respectively; the three EIA kits demonstrated comparable mean % recoveries: 102.9%, 98.3%, and 98.5% for the ELS, CCC, and IDS kits, respectively.

### 3.2. Procedure Selection and Analytical Validation

With regard to recovery measures (virtual or relative), almost all tested combinations of methods led to satisfactory results, showing that GCM can be accurately assayed from fresh or dry African Grey Parrots’ droppings. However, evaluations of the sole influence of extraction buffers (in Section 3.1.1 and Section 3.1.2.) demonstrated remarkable differences between the methods regarding the dynamic range of the assays and the corticosterone recovery from spiked blank extracts: the best procedure showing the broadest dynamic range and the most satisfactory recovery results was a combination of an extraction with 60% methanol and a measurement using the Corticosterone EIA kit from CCC at the working dilutions 1:2 or 1:4. We chose to further validate this procedure using samples that had been subjected to the drying treatment thanks to parallelism and precision evaluations.

**Figure 1:**
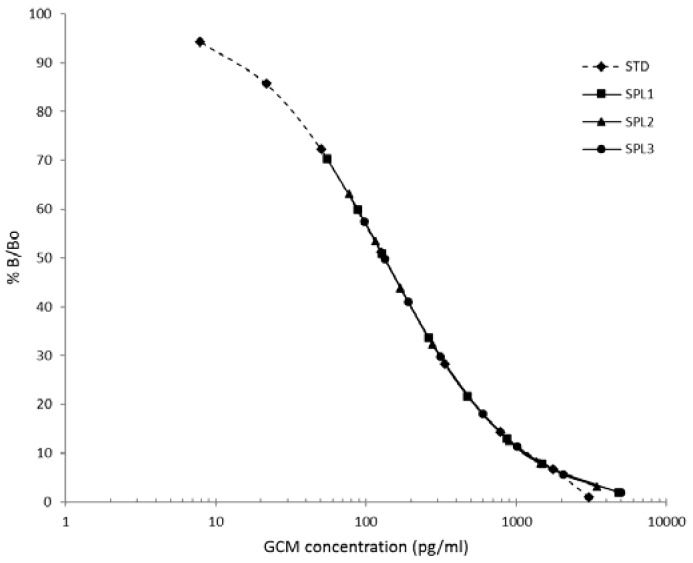
Test for parallelism. The supernatants of three extracts of African Grey Parrots’ droppings (SPL1, SPL2, SPL3) were serially diluted and compared to the standard curve (STD) of the CCC EIA kit. *X*-axis shows the concentration of GCM obtained from the kit in pg/mL. *Y*-axis is the percent of binding (B)/total binding (Bo).

The parallelism study showed that serial dilutions of three samples of dropping extract resulted in a linear decrease in assay values that was parallel to the standard curve (Figure 1). Interestingly, both working dilutions 1:2 and 1:4 are comprised between 50% and 70% of total binding. 

**Figure 2:**
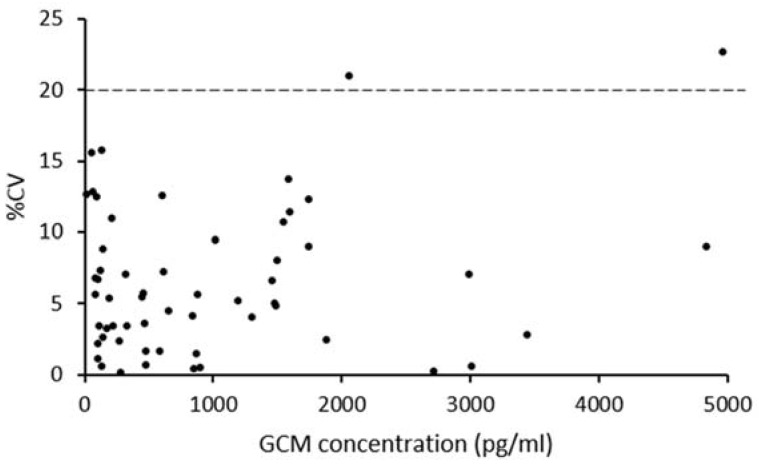
Global precision profile established from the measurements of samples used during the procedure selection and analytical validation of the CCC EIA kit (*n* = 57) showing the obtained intra-assay %CV in function of the GCM concentrations in the EIA wells (pg/mL). The dotted line stands for the 20% limit of acceptable CV%.

Intra-assay %CV obtained from 5 repeated measurements of the QC samples diluted 1:2 were 6.8%, 8.9% and 13.8%, respectively. Figure 2 shows the global precision profile displaying the intra-assay %CV found for each extract sample measured during the previous steps of analytical evaluation, i.e., the procedures carried out for assessing and selecting the best method combination and for completing the analytical validation (parallelism study). All but two samples displayed an acceptable intra-assay %CV inferior to 20%. 

### 3.3. Biological Assessment of the Selected Method

Table 7 shows the descriptive data obtained from the measurements of individual samples from the studied population: dropping GCM concentrations expressed per g of dry droppings (*n* = 31) and HLR counts (*n* = 33). The GCM minimal and maximal concentrations were 26.73 and 107.27 ng/g of dry droppings, respectively, with a mean of 64.01 ± 24.31 ng/g. HLR values ranged from 0.34 to 3.93 (mean = 1.37 ± 0.71).

**Figure 3:**
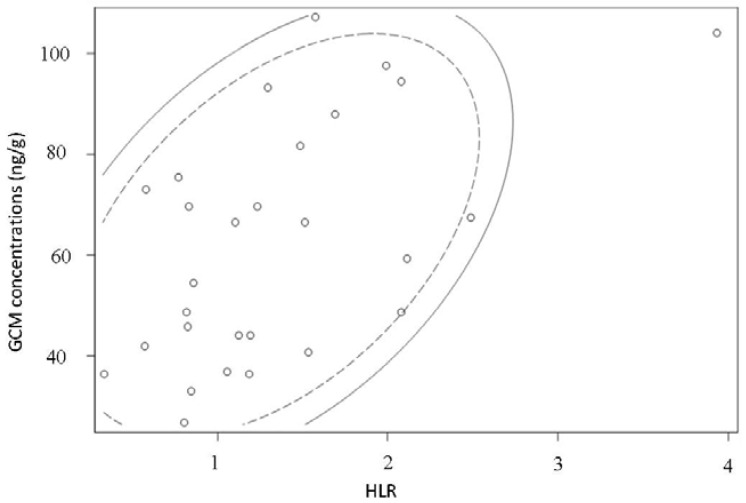
Scatter plot displaying the relationship between individual droppings GCM concentrations (ng/g) and HLR in the studied populations of African Grey Parrots (*n* = 29). The dotted line and the solid line stands for the 70% and 80% prediction ellipses respectively.

Figure 3 shows that a significant positive correlation was found between HLR and GCM concentration on the whole population (Pearson’s correlation coefficient r = 0.48150, *p* = 0.0082, *n* = 29). The dropping dry mass ranged from 0.03 g to 0.40 g (mean = 0.17 ± 0.09 g). The difference between males and females according to dropping mass is not significant (Student *t* test, *t*-value = 1.24, *p* = 0.2245). The difference between male and female GCM levels is not significant (Student *t* test, *t*-value = 0.42, *p* = 0.6782). There was no significant correlation between dropping mass and GCM level (Pearson’s correlation coefficient *r* = −0.30311, *p* = 0.1100, *n* = 29).

## 4. Discussion

This study presents the selection and analytical and biological evaluation of a method to assay GCM in African Grey Parrot droppings. After testing 12 combinations of methods on several analytical validation parameters [22,49], the most accurate method appears to be the combination of an extraction with 60% methanol and measurement using the CCC Corticosterone EIA kit on fresh or dry droppings. Indeed, both pre-treatments resulted in similar measures in this study, as previously observed [50]. However, the global method that went through final validation included the drying of droppings as pre-treatment. Indeed, Palme et al. [47] have highlighted that dried droppings are better suited when samples are small and dry quickly or when undigested materials need to be removed, with both being the case in the present study. The analytical validation was then successfully completed on the whole procedure: accuracy, precision, and parallelism criteria were fulfilled. A biological correlation with stress was demonstrated through a significant and substantial relationship between a reliable stress indicator, HLR, and GCM levels measured in a considerable population of 29 birds. This makes the method described here a feasible and promising tool to assess stress and monitor welfare in the *Psittacus erithacus* species.

Regarding the pre-treatment and extraction procedures, we carefully followed the recommendations of several authors [26,41,44,47], e.g., quickly defrosting samples, homogenization, and high drying temperature. Here, the extraction buffer causing the least interference in the different EIAs we tested was 60% methanol. Extraction buffers based on ethanol or methanol at various percentages are usually employed for extracting GCM in feces or droppings [22,47]. Because of the higher proportion of polar metabolites in bird droppings, we preferred testing a low percentage of alcohol [47]. Many studies (reviewed in [36,45]) measuring GCM in bird droppings or mammalian feces used specially developed EIA with group-specific antibodies or antibodies detecting one specific GCM (such as tetrahydrocorticosterone in Quillfeldt and Möstl [51]). Nevertheless, this approach has already been described as possibly impractical because of the onerous analytical development it requires [45]. We deliberately chose to assess and validate commercially available immunoassays primarily developed to assay corticosterone, but describing many cross-reactions with other glucocorticoids, so that the method could readily be available and accessible for all (e.g., veterinarians in clinics or zoos). There is thus no need to ask for specific antibodies from specialized labs or to fully develop a new specific EIA. The comparison of several immunoassays, including commercial ones, have also been performed by many authors to select the most appropriate assay for measuring GCM in a particular species [50,52,53,54,55].

Each method of GCM measurement should undergo an analytical validation of the assay and a biological evaluation to demonstrate its ability to detect relevant metabolites [22,36,44,56].

The accuracy of the analytical validation was first demonstrated here during the selection of the method through two measures on the droppings: assessment of the corticosterone “virtual recovery” and relative accuracy. The latter analysis was important since it was the only way to estimate the actual recovery of the real GCM. Indeed, the recovery of unmetabolized standard corticosterone in droppings may not correspond with the actual recovery of GCM, as previously highlighted [41,44]. Since the use of radiolabeled and naturally metabolized steroids was precluded in this parrot population from a rescue center, the measure of relative accuracy, i.e., the matching of GCM concentrations obtained from the same sample under a two-fold mass division, was employed to assess the actual recovery of GCM in African Grey Parrot droppings. This approach is often used in immunoassay validation with liquid samples, known as linearity under dilution, and it reflects the accuracy/reliability of the measure [49]. Both virtual recovery and relative accuracy assessments led to satisfactory results (80% < % recovery < 120%) and met the criteria. The analytical validation was completed with a parallelism test, which demonstrated that serial dilutions of the samples resulted in a linear decrease in assay values that was parallel to the standard curve, which evidenced the dose-response relationship [22,44]. Both working dilutions 1:2 and 1:4 fell within the binding range of 50–70%, which is noteworthy as this zone is part of the most linear and reliable standard curve zone (30–70%), according to the kit’s provider. Finally, the analytical validation was achieved through the demonstration of precision using QC samples and a global precision profile: all %CV were satisfactory since they fell below 20% (except two between 20–25%) [49]. By meeting these main criteria (parallelism, accuracy and precision), the selected procedure to assay GCM in African Grey Parrot droppings was analytically validated.

The next step was to biologically assess the assay. A hormonal challenge (ACTH stimulation and/or dexamethasone suppression tests) is typically used [22] to demonstrate the relevancy of the method in evaluating stress. However, the ACTH stimulation induces a potent stress response, which may not reflect the adrenocortical activity that occurs under natural stress conditions [57]. In the present study, ethical and welfare considerations precluded the injection of exogenous substances in the African Grey Parrots of the ASAP center, as it is already a delicate population of an endangered species [36,57]. Popp et al. [58] faced the same issue in a study involving another threatened species, *Amazonia braesiliensis*, and had to prove the biological relevancy of their assay method on another parrot species, *Amazonia aestiva*. Alternatively, the use of a biological stressor on an animal is an acceptable methodology to biologically validate a GCM assay and, in addition, it ensures that the method will appropriately measure GCs in the field when animals are exposed to genuine stressors [22,36]. A study by Ozella et al. [57] used a biological stressor (capture and immobilization) as an effective means to mimic natural stress that causes and elicits genuine levels of GCM released by African Penguins, which demonstrated the advantages of this approach. Here, we developed a new approach to test the biological relevancy in relation to stress assessment, without the injection of exogenous substances: the establishment of a significant correlation with another validated stress indicator, HLR, within the studied population. Indeed, an HLR increase is associated with increased stress levels in all vertebra taxa and particularly in birds, where extensive literature in poultry exists [39]. The stress-induced alteration in the leukocyte profiles is caused by the elevation of glucocorticoids in plasma [40]. HLR is a reliable bioassay of stress because its value is stable and long-lasting and can be directly and positively related to the magnitude of the stressor or to glucocorticoid levels [39]. Of note, the relationship between plasma corticosterone level and HLR has been specifically demonstrated in another parrot species (*Amazona amazonica*) [59]. HLR is increasingly used to assess environmental, social, or physiological stress in wild birds [60,61,62], as well as in parrots [63]. Particularly, previous studies have demonstrated the relevance of this parameter as a physiological indicator of stress in African Grey Parrots [64]. Additionally, practically speaking, HLR evaluation has the advantage of only requiring a drop of blood (5–10 µL) in contrast with the 10-times higher volume needed for a plasma corticosterone assay [39].

Therefore, we chose to correlate GCM levels and HLR, both being outcomes of elevated plasma glucocorticoids over a long-term period (i.e., within hours to days according to the literature [39,40]). The significant and positive substantial relationship between these two long-term stress indicators demonstrates the reliability of the GCM measurement method described here to assess various stress profiles in a sizeable population of African Grey Parrots. The correlation was statistically described as moderate (0.4 < Pearson’s correlation coefficient < 0.7), probably because both the HLR and GCM variables are independently related to a third variable, plasma glucocorticoid level [48], and, furthermore, they did not indicate the same degree of stress response [40]. In addition, the correlation could have been lowered since the glucocorticoid response to stress has been shown to diminish after 85 days of continuous stress, while the HLR response does not decrease over time [40]. Thus, the relationship between the two variables could weaken over time in the case of very long stressful events. This may have been the case in our study population, which was composed of rescued birds with different personal histories. Particularly, with regard to stress, they may have previously faced stressful experiences of various durations, a factor that is impossible to evaluate. However, in spite of these unfavorable factors, the relationship between the data obtained using this GCM measurement method and another well-known and validated stress indicator (HLR) is still described as substantial on a representative bird population (*n* = 29). So, we concluded that the method presented here was biologically relevant. Nevertheless, the authors are aware that a further validation of this method, meaning a physiological validation, should include a hormonal challenge (using ACTH or dexamethasone) in other confirmatory studies on a population of African Grey Parrots where it may be possible, as highlighted by several authors [36,56]. 

Mean GCM basal values differed considerably between the 3 tested EIA in the preliminary step of procedure selection. Such observations have already been made by Stöwe et al. [55], who found significant differences between 4 mean GCM concentrations obtained from four different assays in raven fecal samples, with overlapping ranges extending from 44.37 to 969.41 ng/g. The GCM concentrations measured in our study within the 3 EIA displayed comparable ranges. Furthermore, other authors found similar wide ranges in GCM basal values (31 to 1905 ng/g) analyzed from Blue-Fronted Parrot droppings [65], showing marked individual variations. Other studies on GCM content in parrot droppings from other species described a lower basal range: 6–14 ng/g in Red-Tailed Parrots [58] and 20–110 ng/g in budgerigars [42]. The latter is more like the GCM values found using the EIA (CCC) we finally selected (26.73–107.27 ng/g). In the study’s second step, which including 29 African Grey Parrots droppings, individual variations in GCM levels were also observed, as in Ferreira et al. [65]. These authors suggested that the inter-individual differences they observed could be explained by the previous different life histories of the parrots. That could be particularly true in our case, since the ASAP rescue center purposely welcomes parrots coming from various origins and backgrounds, notably with different stress or welfare histories. Additionally, different coping styles, bacterial gut flora, and individual genetic heterogeneity have been proposed among other factors to explain the commonly observed inter-individual variations [37,66,67]. 

No sex differences in basal GCM concentrations were found in our study, in accordance with other studies in birds [65,68]. Conversely, previous publications often described a sex effect on the GCM levels in birds’ droppings [46,69]. The sex is often described as an important parameter to consider when comparing GCM concentrations [36,37,70], because of possible sex differences in the composition of hormones metabolites as well as the way they are metabolized. In addition, species, seasons, diet and living conditions can also affect the metabolization of glucocorticoid hormones and consequently GCM concentrations in excreta [26,37,71]. In our study, the parrots received the same quality of food ad libitum and were kept in the same housing with identical room temperatures. Moreover, since Ferreira et al. [65] showed that dropping sampling time also matters, all droppings were collected in the same period of the day. As we tried to control the variability of all these parameters (sex, diet, living conditions, sampling time) and keep them constant within the population in our study, this may finally have led to a more similar metabolization process among all the birds and possibly to an absence of sex-effect in the particular species studied here, i.e., *Psittacus erithacus*. It is also possible that the CCC EIA kit, primarily designed to detect corticosterone, was less sensitive to the variation of GCM composition due to sex than other EIAs used previously.

Finally, we did not find any relationship between dropping mass and GCM concentration. This is important since sample mass might bias the study findings [26,72], especially because of possible varied extraction efficiencies and/or very small sample mass. Therefore, we chose to apply a proportional volume of extraction buffer to the sample mass, aiming at maintaining the extraction efficiency independent of dropping mass, as recommended [47]. Also, the dropping samples in our study were all above the critical mass limit described by Millspaugh and Washburn [26], i.e., <0.02 g, which could help prevent the sample mass from influencing the GCM concentration in our study.

## 5. Conclusions

Based on both analytical validation and biological evaluation, we concluded that a combination of an extraction with 60% methanol and measurement using the Cayman Chemical Company Corticosterone EIA kit from dry droppings is a suitable procedure for assaying GCM in African Grey Parrot droppings. The robustness of the method is supported by the absence of relationships between dropping mass and GCM concentration along with the absence of a sex-effect. This relevant method provides a readily available tool to assess stress in African Grey Parrots and opens new perspectives for stress and welfare monitoring in these popular birds kept in captivity in zoos or as pets. The innovative use of another stress indicator, HLR, to biologically assess the method is of great interest. It could be used to assess the reliability of GCM measurement methods in many other species without jeopardizing their welfare through the injection of exogenous substances and creating more stressful situations than those induced by the short restraint involved in collecting a blood drop sample. This advantage is particularly relevant and ethical for individuals coming from an already delicate population since this kind of biological validation is strongly recommended in threatened species, such as *Psittacus erithacus* [57]. 

## Figures and Tables

**Table 1:** Characteristics of commercial corticosterone enzyme immunoassay (EIA) kits tested in this study to assay glucocorticoid metabolites (GCM) in African Grey Parrots’ droppings.

Kit Provider	Enzo Life Sciences	Cayman Chemical	ImmunoDiagnostic Systems
Antibody	Sheep polyclonal corticosterone antibody	Corticosterone specific sheep antiserum	Polyclonal Corticosterone antibody
Detection level	26.99 pg/mL	30 pg/mL	0.55 ng/mL
Compounds and % of cross-reactivity	Deoxycorticosterone 28.6% Progesterone 1.7% Testosterone 0.13% Tetrahydrocorticosterone 0.28% Aldosterone 0.18% Cortisol 0.046% Pregnenolone < 0.03% Estradiol < 0.03% Cortisone < 0.03% 11-dehydrocorticosterone acetate < 0.03%	11-Dehydrocorticosterone 11% Progesterone 7% Cortisol 0.31% Aldosterone 0.17% Testosterone 0.06% Pregnenolone 0.3% 5α-DHT 0.02% Androstenedione 0.1% Cortisone < 0.1% DHEA < 0.1% DHEA-S < 0.1%	11-Dehydrocorticosterone 6.60% 11-Deoxycorticosterone 5.93% Progesterone 1.39% Cortiso l 0.85% Prednisolone 0.60% 21-Deoxycortisol 0.34% 5α-Pregnan-3, 20-dione 0.21% Tetrahydrocortisone < 0.07% Dexamethasone 0.07% DHEA < 0.07% Prednisone < 0.07% Pregnantriol < 0.07% 20β-Hydroxyprogesterone < 0.07% 4-Pregnen-20α-ol-3-one < 0.06% Oestriol < 0.06% Oestradiol < 0.06% Oestrone < 0.06% Pregnenolone < 0.06% 17α-Hydroxypregnolone < 0.05% Cortisone 0.05% Testosterone 0.02% 11-Desoxycortisol 0.02% Aldosterone 0.02% 17α-Hydroxyprogesterone 0.01% Tetrahydrocortisol 0.01%

**Table 2:** Pattern of method combinations and characteristic of samples tested.

Initial Sample (Mass in g) ^a^	Sub-Sample Codes	Pre-Extraction Treatment	Dropping Mass (Fresh or Dry) before Extraction (g)	Extraction Buffer (Volume in mL)
S1 (13.78)	S1DE	Dry	0.84	60% ethanol (8.4)
	S1DM		0.82	60% methanol (8.2)
	S1FE	Fresh	3.44	60% ethanol (34.4)
	S1FM		3.44	60% methanol (34.4)
S2 (7.03)	S2DE	Dry	0.45	60% ethanol (4.5)
	S2DM		0.43	60% methanol (4.3)
	S2FE	Fresh	1.74	60% ethanol (17.4)
	S2FM		1.74	60% methanol (17.4)
S3 ^b^ (7.03)	S3DE	Dry	0.42	60% ethanol (4.2)
	S3DM		0.41	60% methanol (4.1)
	S3FE	Fresh	1.74	60% ethanol (17.4)
	S3FM		1.74	60% methanol (17.4)

^a^ mass S1~2 × mass S2~2 × mass S3; mass S2 = mass S3; ^b^ S3 has been spiked with 0.62 ng of exogenous corticosterone.

**Table 3:** Mean optical density (OD) values (*n* = 3) of EIA dynamic range key parameters (Bo and Blk) in both extraction buffers for each tested commercial kit.

Kit Provider	Enzo Life Sciences				Cayman Chemical				ImmunoDiagnostic Systems			
OD of normal ^a^ Bo	0.880				1.391				1.149			
OD of normal ^a^ Blk	0.170				0.122				0.063			
Extraction buffer	60% ethanol		60% methanol		60% ethanol		60% methanol		60% ethanol		60% methanol	
Working dilution	1:2	1:4	1:2	1:4	1:2	1:4	1:2	1:4	1:2	1:4	1:2	1:4
OD of Bo measured in extraction buffer	0.799	0.936	0.982	0.978	0.747	0.923	1.054	1.178	0.888	0.873	1.052	1.046
OD of Blk measured in extraction buffer	0.174	0.176	0.173	0.173	0.120	0.120	0.118	0.120	0.062	0.060	0.060	0.060

^a^ Normal measures stand for the measures obtained under normal conditions of use of the kits, as recommended by the providers.

**Table 4:** Recovery of corticosterone from spiked blank extract in both extraction buffers in each tested commercial kit.

EIA Kit	Extraction Buffer	Working Dilution	Expected ^a^ Corticosterone Concentration (ng/mL)	Measured Corticosterone Concentration (ng/mL)	Recovery (%)
ELS	60% ethanol	1:2	0.80	0.09	11
		1:4		0.10	13
	60% methanol	1:2		<0.03	ND
		1:4		0.11	13
CCC	60% ethanol	1:2	0.13	0.32	258
		1:4		0.21	170
	60% methanol	1:2		0.13	101
		1:4		0.11	88
IDS	60% ethanol	1:2	16.40	8.21	50
		1:4		14.71	89
	60% methanol	1:2		4.16	25
		1:4		11.68	71

ND: Not determined. ^a^ Expected corticosterone concentrations were calculated based on the added amount of standard corticosterone in each buffer, taking in account the dilution factor.

**Table 5:** Virtual recovery of corticosterone in African Grey Parrots’ droppings under all tested conditions.

EIA Kit	Sub-Sample Code (*n* = 2 or 3)	Working Dilution	Measured GCM Concentration in S2 (ng/g Droppings)	Expected GCM Concentration in S3 (ng/g Droppings)	Measured GCM Concentration in S3 (ng/g Droppings)	Recovery (%)
ELS	SnDE	1:2	34.00	34.62	29.15	84
		1:4	56.14	56.76	47.70	84
	SnFE	1:2	30.48	31.10	29.79	96
		1:4	47.93	48.55	47.13	97
	SnDM	1:2	47.17	47.79	58.73	123 ^a^
		1:4	79.53	80.15	99.59	124 ^a^
	SnFM	1:2	29.02	29.64	25.65	87
		1:4	64.04	64.66	50.55	78 ^a^
CCC	SnDE	1:2	ND	ND	104.61	ND
		1:4	76.67	77.29	64.9	84
	SnFE	1:2	143.55	144.17	138.77	96
		1:4	99.14	99.76	97.69	98
	SnDM	1:2	59.62	60.24	66.00	110
		1:4	52.25	52.87	65.12	123 ^a^
	SnFM	1:2	66.59	67.21	76.56	114
		1:4	71.78	72.40	72.65	100
IDS	SnDE	1:2	582.23	582.85	488.73	84
		1:4	485.71	486.33	485.30	100
	SnFE	1:2	898.81	899.43	1063.46	118
		1:4	684.06	684.68	702.36	103
	SnDM	1:2	368.91	369.53	408.95	111
		1:4	337.61	338.23	360.84	107
	SnFM	1:2	454.38	455.00	440.72	97
		1:4	185.02	185.64	219.13	118

ND: Not determined, because of OD values out of the standard curve. ^a^ Not satisfactory % recovery since out of the acceptable range 80–120% for immunoassays.

**Table 6:** Relative accuracy of GCM measurements under all tested conditions.

EIA Kit	Sub-Sample Code (*n* = 1 or 2)	Working Dilution	Measured GCM Concentration in S1 (ng/g Droppings)	Measured GCM Concentration in S2 (ng/g Droppings)	Recovery (%)
ELS	SnDE	1:2	34.76	34.00	102
		1:4	52.83	56.14	94
	SnFE	1:2	28.77	30.48	94
		1:4	52.41	47.93	109
	SnDM	1:2	47.98	47.17	102
		1:4	79.71	79.53	100
	SnFM	1:2	34.96	29.02	120
		1:4	65.15	64.04	102
CCC	SnDE	1:2	ND	ND	ND
		1:4	67.76	76.67	88
	SnFE	1:2	125.74	143.55	88
		1:4	90.66	99.14	91
	SnDM	1:2	65.67	59.62	110
		1:4	57.09	52.25	109
	SnFM	1:2	69.35	66.59	104
		1:4	70.56	71.78	98
IDS	SnDE	1:2	569.91	582.23	98
		1:4	470.51	485.71	97
	SnFE	1:2	1048.88	898.81	117
		1:4	601.18	684.06	88
	SnDM	1:2	376.34	368.91	102
		1:4	334.55	337.61	99
	SnFM	1:2	430.53	454.38	95
		1:4	170.15	185.02	92

ND: Not determined, because of OD values out of the standard curve.

**Table 7:** Sex, Heterophil: Lymphocyte Ratio (HLR), and individual droppings’ data of the African Grey Parrot studied population.

Parrot Code	Sex	HLR	Mass of Dry Droppings (g)	GCM Concentration (ng/g)
G1	M	0.99	0.22	ND
G2	M	1.12	0.27	44.25
G3	M	1.22	NC	ND
G4	M	1.53	0.11	40.80
G5	M	2.08	0.18	94.50
G6	M	0.82	0.12	48.79
G7	M	0.58	0.16	73.00
G8	F	1.49	0.38	81.63
G9	M	1.19	0.15	44.05
G10	M	1.19	0.40	36.53
G11	F	1.30	0.18	93.38
G12	F	1.11	0.03	66.43
G13	M	2.49	0.11	67.43
G14	F	1.23	0.20	69.64
G15	M	0.34	0.11	36.41
G16	M	0.86	0.12	54.37
G17	M	2.19	0.03	ND
G18	M	1.51	0.06	66.43
G19	F	1.06	0.33	36.78
G20	F	2.03	NC	ND
G21	F	0.85	0.19	33.08
G22	M	3.93	0.06	104.21
G23	M	0.90	0.22	104.29
G24	F	2.08	0.26	48.67
G25	F	0.81	0.24	26.73
G26	F	0.83	0.14	45.91
G27	F	1.58	0.07	107.27
G28	F	0.83	0.19	69.58
G29	F	1.69	0.14	88.09
G30	M	0.57	0.14	41.93
G31	F	1.99	0.09	97.64
G32	F	2.12	0.29	59.19
G33	M	0.77	0.15	75.33

M: Male; F: Female; NC: Not Collected; ND: Not Determined; in two cases, the signal measured was out of the standard curve of the assay and in the two other cases, the assay could not be performed in absence of droppings (NC).
